# Hypoalbuminemia as a prognostic marker for survival in biliary tract cancer: associations with tumor type, treatment and sex

**DOI:** 10.1007/s00432-025-06408-2

**Published:** 2026-01-08

**Authors:** Mostafa Abdulrazzak, Linda Björkhem-Bergman, Christina Villard, Marco Gerling, Tina Gustavell, Hannes Jansson

**Affiliations:** 1 Division of Surgery and Oncology, C1 77, Department of Clinical Science, Intervention and Technology, Karolinska Institutet, Karolinska University Hospital, 141 86 Stockholm, Sweden; 2 Division of Clinical Geriatrics, Department of Neurobiology, Care Sciences and Society, Karolinska Institutet, Stockholm, Sweden; 3 Palliative Medicine, Stockholms Sjukhem, Mariebergsgatan 22, Stockholm, Sweden; 4 Division of Transplantation Surgery, Department of Clinical Science, Intervention and Technology, Karolinska Institutet, Karolinska University Hospital, Stockholm, Sweden; 5 Department of Upper Abdominal Diseases, Karolinska University Hospital, Stockholm, Sweden; 6 Division of Nursing, Department of Neurobiology, Care Sciences and Society, Karolinska Institutet, Stockholm, Sweden

**Keywords:** Biliary tract cancer, Cholangiocarcinoma, Gallbladder cancer, Prognostic factors, Surgery, Sex differences

## Abstract

**Background:**

Albumin in combination with other inflammatory markers has shown prognostic value in malignancy, including biliary tract cancer (BTC). This study aimed to evaluate the prognostic value of hypoalbuminemia alone in patients with BTC, with stratified analyses according to tumor type, treatment and sex.

**Methods:**

A retrospective regional referral center cohort study was conducted, including consecutive patients with a measurement of preoperative albumin and intended resection of suspected BTC: intrahepatic cholangiocarcinoma (iCCA), perihilar cholangiocarcinoma (pCCA) or gallbladder cancer (GBC) between 2009 and 2017. The primary outcome was overall survival (OS), analyzed by Kaplan-Meier estimate and Cox regression.

**Results:**

Out of 221 patients, 191 underwent resection, while 30 patients were diagnosed with unresectable BTC (14%). In the resection group, 147 patients had confirmed BTC, while 44 (20%) were postoperatively diagnosed with a benign lesion. Hypoalbuminemia (< 35 g/L) was more frequent in pCCA (75%) and GBC (59%), compared to iCCA (34%, *p* < 0.001). The preoperative albumin level was positively associated with resectability (*p* = 0.025). In patients with resection, hypoalbuminemia was associated with a tumor positive resection margin (*p* < 0.001). Hypoalbuminemia was a negative prognostic factor in resectable (*p* < 0.001) and unresectable BTC (*p* < 0.001), and in both women (*p* = 0.002) and men (*p* = 0.004). Hypoalbuminemia was negatively associated with OS in iCCA (*p* < 0.001) and GBC (*p* = 0.022), but not in pCCA (*p* = 0.210).

**Conclusion:**

Preoperative albumin was prognostic for survival in patients with iCCA and GBC, in both women and men and regardless of tumor resectability. Patients with pCCA more often had low albumin, and hypoalbuminemia alone was not prognostic in this subgroup.

**Supplementary Information:**

The online version contains supplementary material available at 10.1007/s00432-025-06408-2.

## Introduction

Biliary tract cancers (BTC ) are a group of epithelial malignancies originating from the biliary ducts, comprising cholangiocarcinoma (CCA) and gallbladder cancer (GBC). According to anatomical subtype, CCA can be classified as intrahepatic (iCCA), perihilar (pCCA) or distal cholangiocarcinoma (dCCA) (Banales et al. 2025). These cancers are generally associated with a poor prognosis, in part explained by late diagnosis and with a high risk of recurrence after curative intent treatment (Banales et al. 2025, Balakrishnan et al. 2023). The only available curative approach is surgery, i.e. radical resection, or for selected patients with localized CCA liver transplantation (Ethun et al. 2018, European Association for the Study of the Liver 2023). Adjuvant oncological therapy has only recently been established as a standard of care after resection (Primrose et al. 2019, Nakachi et al. 2023). For patients with advanced and unresectable tumors, non-curative tumor-directed treatment can include systemic and locoregional oncological therapy (Izquierdo-Sanchez et al. 2022).

Prognostication in cancer disease is important, as it can influence clinical decision-making and inform the selection of the most optimal therapeutic choices among available treatment options. Importantly, prognostic information may also affect a patient’s decisions and priorities (Mack et al. 2015, van der Velden et al. 2022). Plasma albumin concentration in combination with other inflammatory markers such as C-reactive protein (CRP) or lymphocyte count has shown prognostic value in different types of malignancy (Dolan et al. 2018), including BTC (Jansson et al. 2020). Albumin alone has been proposed as a biomarker of cachexia (Goodrose-Flores et al. 2022), with a negative prognostic value in advanced cancer and an association with cancer-related symptoms (Goodrose-Flores et al. 2022, Stares et al. 2021).

Importantly, albumin concentrations differ between women and men (Weaving et al. 2016), and sex-differences have been identified with regard to the association between plasma albumin and cancer-related symptoms (Goodrose-Flores et al. 2022). Whether the prognostic value of hypoalbuminemia differs depending on sex or disease stage remains to be determined.

The aim of the present study was to evaluate the prognostic value of hypoalbuminemia in patients with suspected BTC, with stratification of analysis according to tumor type, treatment (resection or non-resection) and sex. To this end, a cohort of patients planned for curative intent surgery for suspected BTC was analyzed to assess prognosis in relation to preoperative albumin levels.

## Methods

### Study cohort

A retrospective analysis was performed based on a cohort of consecutive patients undergoing surgical exploration for suspected BTC at the Stockholm County regional referral center (Karolinska University Hospital, Stockholm, Sweden), from 1 January 2009 to 31 May 2017. Included were adult patients with an available preoperative measurement of plasma albumin, taken the day before surgery or at the last preoperative assessment. The study cohort consisted of patients with resected BTC, patients with BTC found unresectable at exploration, as well as patients with suspected BTC and a postoperative finding of benign lesions. The study was conducted in accordance with the declaration of Helsinki (World Medical Association 2025) and approved by the Regional Ethical Review Board and the Swedish Ethical Review Authority (2013/188-31/1, 2020-02702, 2015/259 − 31/2, 2022-06962-02). The study was reported in accordance with the Strengthening the Reporting of Observational Studies in Epidemiology (STROBE) guidelines (Vandenbroucke et al. 2007). The STROBE checklist is provided in Supplementary Table 1.

## Outcomes and clinicopathological data

The primary outcome variable was overall survival (OS), measured from the date of surgery. Secondary outcome variables were disease-free survival (DFS) and postoperative complications according to the Clavien-Dindo classification (Dindo et al. 2004). Demographic and clinicopathological variables included age, sex, American Society of Anesthesiologist physical status classification (ASA) (Mayhew et al. 2019), plasma albumin concentration (g/L), plasma c-reactive protein (CRP) concentration (mg/L), plasma carbohydrate antigen 19 − 9 (CA19-9) concentration (kU/L), prothrombin time - international normalized ratio (PT-INR), type of surgical resection (major resection defined as hepatic resection including three or more liver segments ), tumor stage according to the 7th edition of TNM/AJCC guidelines and tumor grade according to the College of American Pathologists (Burgart et al. 2021, Sobin et al. 2010). Hypoalbuminemia was defined as a plasma albumin level below 35 g/L (Gatta et al. 2012). Data were collected from the electronic health records, linked to the Swedish National Population Registry for data on vital status, and local quality registries.

### Statistical analysis

Continuous variables were presented as medians and interquartile ranges (IQR), with non-parametric comparisons using the Mann-Whitney U test. For data following a Gaussian distribution, two-sided, unpaired t-tests were employed to assess intergroup differences. Categorical variables were presented as numbers and percentages. The chi-square and Fisher’s exact tests were used, as appropriate, for the comparison of categorical variables. Survival analysis was performed by the Kaplan-Meier method and Cox regression. The proportional hazards assumption was tested with time-dependent covariates. The discriminatory ability of prognostic markers was assessed with Harrell’s concordance index (c-index), where a c-index of 0.50 indicates no predictive ability and a c-index of 1.00 would denote perfect predictive ability (Harrell 2015). Median follow-up time was calculated according to the reverse Kaplan-Meier method. Statistical analyses were performed with SPSS Statistics version 28 and 30 (IBM, Armonk, NY, USA), GraphPad Prism version 9 (GraphPad, San Diego, CA, USA) and RStudio version 2025.09.2 + 418 (Posit Software, PBC, Boston, MA, USA), with the survival package (Therneau and Grambsch 2000). A two-sided p-value < 0.05 was considered as statistically significant.

## Results

### Demographic and clinicopathological data

**Fig. 1:**
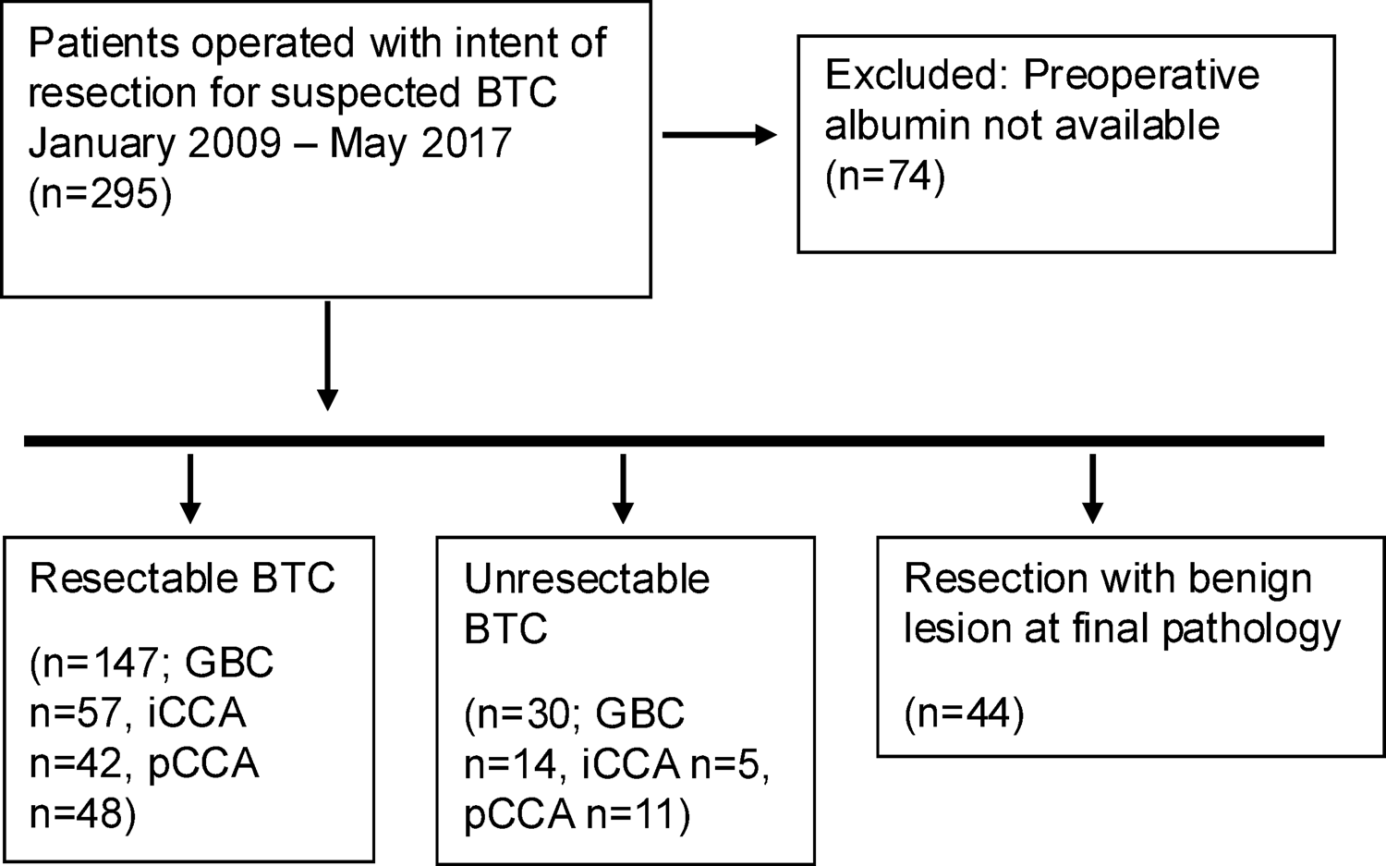
Study inclusion flow chart. BTC: biliary tract cancer, GBC: gallbladder cancer, iCCA: intrahepatic cholangiocarcinoma, pCCA: perihilar cholangiocarcinoma

The study flow chart is presented in Fig. 1. Out of 295 patients undergoing surgery, data on the preoperative plasma albumin level was available for 221 patients, 117 women (53%) and 104 men (47%), who were included for analysis. One-hundred and ninety-one patients (86%) underwent resection with curative intent, while 30 patients (14%) were diagnosed with unresectable BTC at exploration. In the group of patients undergoing resection, 147 patients (77%) had postoperatively confirmed BTC, whereas 44 patients (23%) were postoperatively diagnosed with benign lesions according to final histopathology. A total of 177 patients had histologically confirmed BTC.

Preoperative biliary drainage was performed for 85 patients with BTC (48%), most often by the endoscopic route (endoscopic drainage *n* = 55, percutaneous transhepatic biliary drainage *n* = 6, combination of endoscopy and percutaneous drainage *n* = 24). While biliary drainage was performed for a majority of patients with pCCA (*n* = 53, 90%), it was less often necessary for patients with GBC (*n* = 28, 39%) or iCCA (*n* = 4, 9%).

The median follow-up time was 57 months (IQR 47–77 months). The median OS time was 25 months (95% CI 18–32 months) for patients with BTC, while for patients with benign lesions the median OS was not reached. Demographic and clinical characteristics for patients with BTC (*n* = 177), stratified according to presence or absence of hypoalbuminemia (albumin < 35 g/L), are presented in Table 1.

### Albumin levels

**Fig. 2:**
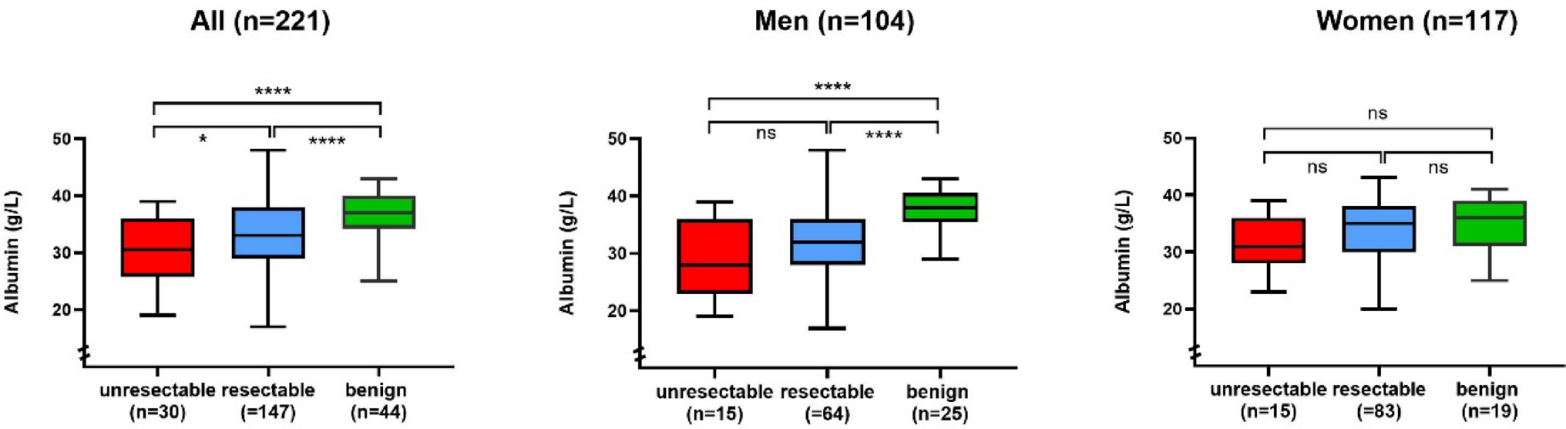
Preoperative albumin levels in patients with biliary tract cancer (red = unresectable, blue = resectable) and benign lesions (green). Two-sided, un-paired t-test. *p < 0.05, ****p < 0.0001

The median albumin levels were 33 g/L (IQR 29–38) in patients with resectable BTC, 31 g/L (IQR 26–36) in patients with unresectable BTC and 37 g/L (IQR 34–40) in patients with benign lesions. The preoperative albumin level was significantly lower in patients with confirmed malignancy compared to patients with benign lesions (Fig. 2, *p* < 0.001). The difference in albumin levels between patients with BTC and patients with benign lesions was found to be significant in men, but not in women (Fig.  2, men *p* < 0.001, women *p* = 0.198). In patients with BTC (*n* = 221), the preoperative albumin concentration was associated with tumor resectability at the group level (Fig. 2, *p* = 0.025), but not in subgroup analysis according to gender.

Among patients with BTC, hypoalbuminemia (< 35 g/L) was more frequent in patients with pCCA (75%) and GBC (59%) compared to patients with iCCA (34%, *p* < 0.001, Table 1). In patients with BTC, hypoalbuminemia was more frequent among men than among women (67% vs. 50%, *p* = 0.022). In patients with resection, hypoalbuminemia was associated with a microscopically tumor positive resection margin (*p* < 0.001), but not with the extent of resection (*p* = 0.727). Hypoalbuminemia was also associated with preoperative biliary drainage (*p* < 0.001), the preoperative bilirubin level (*p* < 0.001), the preoperative CRP level (*p* < 0.001) and an increased CA19-9 (*p* < 0.001). A majority of patients with BTC had hypoalbuminemia (*n* = 102, 58%), and the rate of hypoalbuminemia was similar in patients with and without resection (Table 1). While the albumin level (g/L) was associated with tumor resection (Fig. 2, *p* = 0.025), no association to resection was seen for hypoalbuminemia (Table 1, *p* = 0.272).

## Survival analysis

On univariable analysis, hypoalbuminemia was a negative prognostic factor for OS both in patients with unresectable and resectable BTC; hazard ratio (HR) 2.56 (95% confidence interval [CI] 1.05–6.22, *p* = 0.038); and HR 2.24 (95% CI 1.49–3.37, *p* < 0.001) respectively. Hypoalbuminemia remained prognostic regardless of sex; HR 2.15 (95% CI 1.32–3.51, *p* = 0.002) in women and HR 2.35 (95% CI 1.32–4.16, *p* = 0.004) in men.

**Fig. 3:**
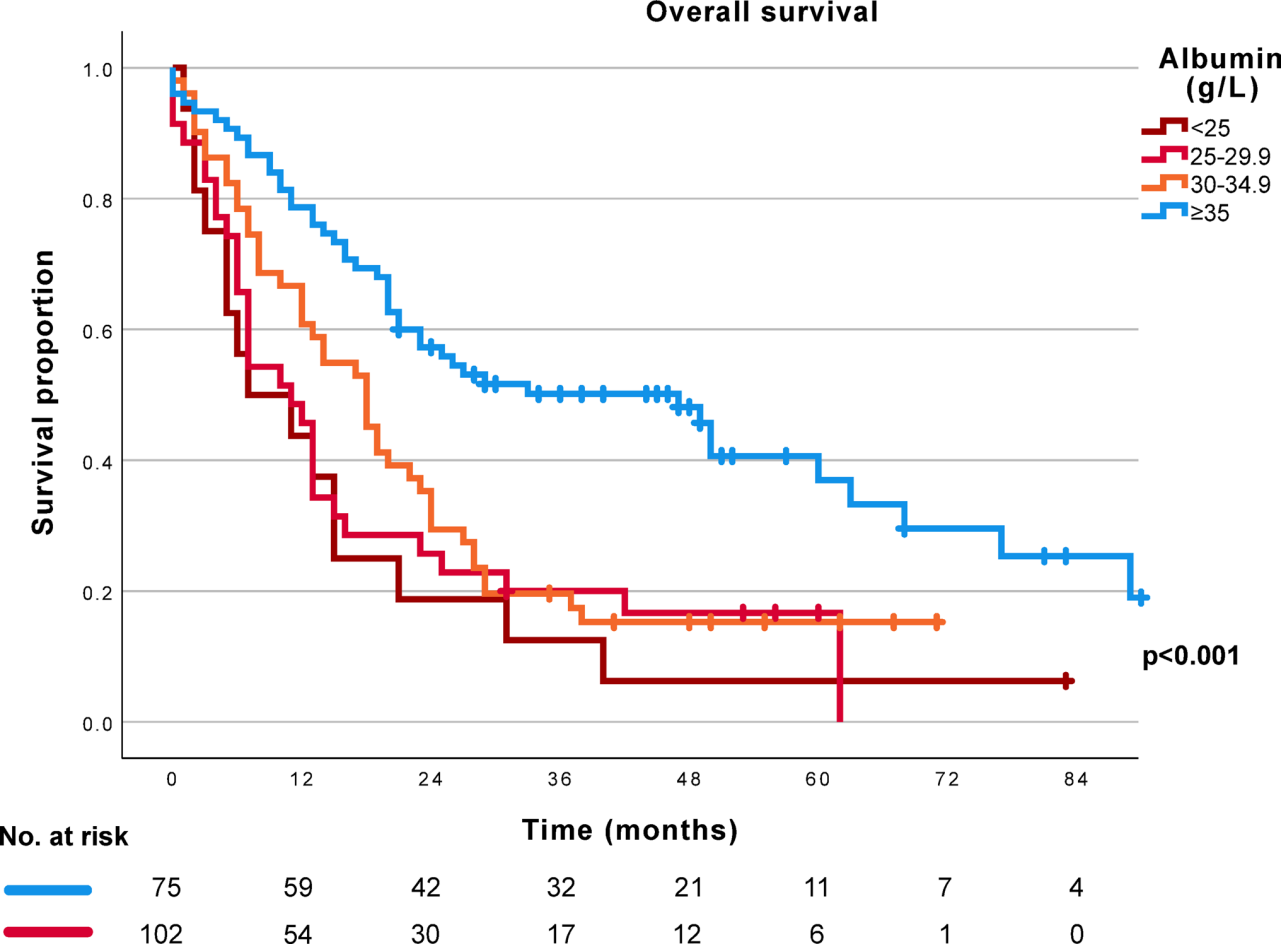
Overall survival for patients with biliary tract cancer according to preoperative albumin levels, patients with normal albumin in blue

Survival curves for patients with BTC are presented in Fig. 3, with stratification according to the preoperative albumin level. While patients with a normal preoperative albumin had a median OS of 47 months (95% CI 28–66 months), patients with preoperative hypoalbuminemia had a median OS of 13 months (95% CI 10–16 months, *p* < 0.001). No significant OS differences were seen within the hypoalbuminemia group on further stratification according to the degree of hypoalbuminemia (<25 g/L vs. 25–29.9 g/L *p* = 0.579, < 25 g/L vs. 30–34.9 g/L *p* = 0.138, 25–29.9 g/L vs. 30–34.9 g/L *p* = 0.319, Fig. 3).

Within the subgroup of BTC patients undergoing major hepatic resection (*n* = 95), median OS was 49 months for patients with normal albumin (95% CI 25–73 months) and 13 months for patients with hypoalbuminemia (95% CI 10–16 months, *p* < 0.001) (Supplemental Fig. 1).

While the proportion of patients with recurrence after resection did not differ between patients with and without hypoalbuminemia (Table 1), there was a significant difference in time to recurrence. The median DFS was 12 months (95% CI 7–17 months) for patients with hypoalbuminemia, compared to 18 months (95% CI 11–25 months, *p* = 0.022, Supplemental Fig. 2) for patients with normal albumin.

Survival curves for patients with unresectable BTC are presented in Supplemental Fig. 3, revealing significantly decreased survival (*p* = 0.026) for patients with hypoalbuminemia. Median OS was 6 months (95% CI 5–7 months) in patients with hypoalbuminemia and 11 months (95% CI 2–20 months) in patients with a normal preoperative albumin.

In subgroup analyses according to the type of BTC, hypoalbuminemia was prognostic for overall survival in patients with iCCA (HR 5.79, 95% CI 2.54–13.21, *p* < 0.001) and GBC (HR 1.89, 95% CI 1.09–3.30, *p* = 0.024), but not in patients with pCCA (HR 1.54, 95% CI 0.78–3.05, *p* = 0.213).

**Table 1:** Demographic and clinical characteristics of patients with biliary tract cancer (*n* = 177), with stratification according to presence or absence of hypoalbuminemia

Variable	Albumin ≥ 35 g/L (*n* = 75)	Albumin < 35 g/L (*n* = 102)	*p*-value
Diagnosis			**< 0.001**
pCCA, n (%)	15 (20)	44 (43)	
iCCA, n (%)	31 (41)	16 (16)	
GBC, n (%)	29 (39)	42 (41)	
Age, years, median (IQR )	65 (55–71)	66 (58–71)	0.459
Sex			**0.022**
Women, n (%)	49 (67)	49 (48)	
Men, n (%)	26 (33)	53 (52)	
ASA ≥ 3 (yes ), n (%)	24 (32)	34 (33)	0.852
PSC (yes ), n (%)	9 (12)	9 (9)	0.490
Biliary drainage (yes ), n (%)	19 (25)	66 (65)	**< 0.001**
Bilirubin, micromole/L (missing data *n* = 2), median (IQR )	6 (5–10)	10 (6–21)	**< 0.001**
CA 19 − 9 ≥ 35 kU/L (missing data *n* = 83), n (%)	15 (36)	42 (81)	**< 0.001**
CRP, mg/L, median (IQR )	5 (2–9)	15 (5–35)	**< 0.001**
PT-INR (missing data *n* = 4), median (IQR )	1.00 (1.00-1.05)	1.00 (1.00-1.10)	0.086
Resection (yes ), n (%)	65 (87)	82 (80)	0.272
Major resection (yes ), n (%)^#^	41 (63)	54 (66)	0.727
Postoperative complication (CD ≥ 3) (missing data *n* = 1), n (%)^#^	27 (42)	42 (52)	0.215
T ≥ 3 (missing data *n* = 1), n (%)^#^	27 (42)	45 (56)	0.092
N1 (missing data *n* = 26), n (%)^#^	29 (57)	36 (51)	0.554
Pn1 (missing data *n* = 25), n (%)^#^	40 (73)	52 (78)	0.533
LV1 (missing data *n* = 16), n (%)^#^	45 (76)	59 (82)	0.424
R1 (missing data *n* = 4), n (%)^#^	30 (46)	61 (78)	**< 0.001**
Adjuvant chemotherapy (missing data *n* = 20), n (%)^#^	4 (7)	4 (6)	1.000^§^
Recurrence (yes ) (missing data *n* = 24), n (%)^#^	38 (67)	51 (77)	0.190
Tumor specific treatment at recurrence (missing data *n* = 32), n (%)^#^	28 (74)	29 (60)	0.180

ASA: American Society of Anesthesiologists physical status class, CRP: c-reactive protein, LV1: lymphovascular invasion, N1: lymph node metastasis, Pn1: perineural invasion, PSC: primary sclerosing cholangitis, PT-INR: prothrombin time - international normalized ratio, R1: microscopically tumor-positive resection margin, T ≥ 3: tumor extension 3–4

§ Fisher’s Exact test. P-values < 0.05 in bold. # Reported for patients undergoing resection

Univariable and multivariable Cox regression analyses for patients with BTC are presented in Table 2. Tumor extension status, lymph node metastasis, preoperative albumin, CRP, PT-INR and tumor resectability were associated with survival on univariable analysis. The highest predictive values, as assessed by c-index, were seen for tumor extension status, followed by albumin and CRP (c-index 0.65, 0.64 and 0.63, respectively). In multivariable analysis, preoperative inflammatory markers albumin and CRP were both independent prognostic factors, together with tumor extension and preoperative PT-INR.

**Table 2:** Uni- and multivariable Cox regression analyses for overall survival in BTC

Variable:	Univariable analysis HR (95% CI)	*p*-value	c-index	Multivariable analysis HR (95% CI)	*p*-value
ASA ≥ 3 *	1.02 (1.00-1.03)	0.064		1.02 (1.00-1.04)	0.070
Age	1.01 (0.99–1.02)	0.370		1.00 (0.98–1.02)	0.866
Sex (female )	0.73 (0.52–1.02)	0.065		1.05 (0.64–1.73)	0.850
BMI	0.99 (0.96–1.03)	0.647		0.98 (0.93–1.03)	0.351
T ≥ 3	2.65 (1.84–3.80)	**< 0.001**	0.65	1.87 (1.17-3.00)	**0.009**
N1	1.62 (1.08–2.45)	**0.021**	0.56	1.53 (0.96–2.43)	0.071
Preoperative albumin level (g/L)	0.94 (0.91–0.97)	**< 0.001**	0.64	0.96 (0.92-1.00)	**0.028**
Preoperative CRP level (mg/L)	1.01 (1.01–1.02)	**< 0.001**	0.63	1.01 (1.00-1.02)	**0.019**
Preoperative PT-INR	5.52 (1.60-19.08)	**0.007**	0.56	19.4 (2.92-128.85)	**0.002**
Preoperative bilirubin (micromole/L)	1.00 (1.00-1.01)	0.233		1.00 (0.99–1.01)	0.413
Resection (yes )	0.36 (0.23–0.55)	**< 0.001**	0.57	0.68 (0.30–1.51)	0.340

ASA: American Society of Anesthesiologists physical status class, BMI: body mass index, BTC: biliary tract cancer, c-index: concordance index, LV1: lymphovascular invasion, N1: lymph node metastasis, PT-INR: prothrombin time - international normalized ratio, T ≥ 3: tumor extension 3–4

*: as time-dependent variable. P-values < 0.05 in bold

As both albumin and CRP maintained a predictive value for OS in multivariable analysis, the combined CRP-to-albumin ratio was assessed, with a significant association to OS (HR 1.04, 95% CI 1.02–1.05, *p* < 0.001) and a predictive ability similar to that of either marker alone (c-index 0.64).

## Discussion

This study reports prognostic associations for preoperative albumin in a clinical cohort of patients with resectable and unresectable BTC. Both in patients with resectable BTC and in patients with BTC found unresectable, hypoalbuminemia was a strong negative prognostic factor. In fact, in the multivariable analysis, the albumin level and tumor extension were independent prognostic factors, rather than resectability. This underlines how tumor status/-biology, and systemically measurable responses to tumor growth, are central prognostic factors. A large proportion of patients in this study had GBC, a malignancy where recent data have indicated a limited value of up-front resection surgery for patients with locally advanced tumors (Balakrishnan et al. 2023). Importantly, in the current study, collinearity between tumor extension, albumin levels and resection status must be considered when interpreting results from the multivariable regression.

This is, to our knowledge, the first study to explore possible sex-differences in albumin as a prognostic factor. This is of relevance as previous research has suggested sex-differences in the association between albumin and cancer symptoms. A recent study investigating appetite in late-stage cancer patients indicated that albumin levels were associated with loss of appetite in men but not in women (Goodrose-Flores et al. 2022). In the current study, we could show that albumin levels were prognostic in both women and men. While the preoperative albumin levels differed significantly between patients with BTC and patients with benign lesions, this association between low albumin and malignancy was only statistically significant in men. One contributing factor was that hypoalbuminemia in BTC was found to be less common in women than in men. Furthermore, normal albumin levels have been shown to be lower in women than in men (Weaving et al. 2016), possibly contributing to the less pronounced and statistically not significant difference in plasma albumin between women with benign lesions and women with BTC in this cohort. While albumin traditionally has been evaluated as a marker of nutritional status, recent research has underscored the role of low albumin as a marker for systemic inflammation, both in acute and chronic conditions, including malignancy (Nazha et al. 2015, Alves et al. 2018, Friedman & Fadem 2010).

The rate of adjuvant oncological treatment was low in this cohort, as the study period preceded results from the BILCAP trial (Primrose et al. 2019) and the clinical implementation of postoperative capecitabine as a standard of care. Hypoalbuminemia is a known negative prognostic factor in advanced malignancy and can be part of a decision not to initiate systemic chemotherapy. However, in this study no significant differences were seen in the rates of tumor-specific treatment of recurrence between patients with and without preoperative hypoalbuminemia before primary resection.

While hypoalbuminemia was associated with a tumor-positive resection margin, no association was seen with the extent of resection. This finding is in line with the understanding of a microscopically non-radical resection as a factor related to the local tumor extension and tumor growth pattern, rather than a purely technical, surgery-related factor (Demir et al. 2018). A decreased albumin level has been interpreted as a possible consequence of tumor progression and a biomarker of cachexia (Goodrose-Flores et al. 2022, Stares et al. 2021, Lipshitz et al. 2024), rather than low albumin having any direct causative effects on prognosis.

In pancreatic cancer, preoperative prognostic scores have recently been suggested as one way to improve the preoperative risk stratification and to define a concept of biological borderline resectability, supporting consideration of neoadjuvant therapy (Oba et al. 2022, Bockhorn et al. 2014). To which degree a poor prognostic marker reflects tumor factors (such as micrometastasis) and/or host factors (such as a non-effective immune response) is not clear. Likewise, whether a poor long-term prognosis in BTC can be improved by neoadjuvant oncological treatment, and how the prognostic value of albumin is affected by systemic therapy, remains to be determined. Results from the current study support that albumin could be included in preoperative prognostic scores for patients with iCCA and GBC, while for patients with pCCA – where biliary obstruction and preoperative interventions are more frequent – the isolated prognostic value of albumin levels could be diminished by the presence of cholestasis and infection. Sequential assessment of albumin and other inflammatory markers has been proposed to be included in future clinical trials (Dolan et al. 2018), which could provide prospective validation to support implementation of prognostic scores for patients with different types of BTC.

Several important variables were unavailable in this study, including performance status classification according to the World Health Organization/Eastern Cooperative Oncology Group (Oken et al. 1982) and patient-reported symptoms. With previous research having indicated a prognostic impact of decreased appetite and fatigue (Goodrose-Flores et al. 2022), patient symptoms as well as functional status could be an important tool for further risk stratification. Furthermore, data on preoperative oncological therapy was lacking in the study database. Neoadjuvant therapy for resectable BTC has not been used in local clinical routine in the study period. Only rare patients with initial systemic therapy achieving downstaging or stable disease have been operated. For patients with pCCA, including a more recent time period at the same center, the rate of preoperative chemotherapy was reported at 3% (Jansson et al. 2025).

Strengths of this study include the long follow-up time and a regional cohort with complete survival data. Limitations include the single-center design with a limited sample size restricting statistical power, loss to follow-up with regard to recurrence data, a study period preceding implementation of adjuvant therapy as standard of care, and a cohort restricted to patients assessed as candidates for resection surgery. The later aspect means that patients with unresectable BTC in this analysis only represent a subgroup of the population of patients with advanced BTC. However, this analysis should be of value for our understanding of systemic inflammation as a prognostic factor in patients undergoing evaluation for curative intent surgery.

In conclusion, this study found preoperative albumin to be a prognostic marker of survival in patients with iCCA and GBC, in both women and men and regardless of tumor resectability. Patients with pCCA more often had low albumin, and hypoalbuminemia alone was not prognostic in this subgroup.

## Supplementary Information

Below is the link to the electronic supplementary material.

Supplementary Material 1

## Data Availability

The data supporting the findings of this study are available from the corresponding author on reasonable request.
